# Comparison of the Effect of Proanthocyanidin Surface Treatments on Shear Bond Strength of Different Cements

**DOI:** 10.3390/ma12172676

**Published:** 2019-08-22

**Authors:** Şifa Atabek, A. Nehir Özden

**Affiliations:** Department of Prosthodontics, Faculty of Dentistry, Near East University, Lefkosa, 99138 Mersin 10, Turkey

**Keywords:** grape seed extract, dental cements, shear strength

## Abstract

This study aimed to compare the effect of proanthocyanidin-rich grape seed extract (Pa-rich GSE) in two different concentrations on the bond strength to dentin tissue for four different cement groups (resin cement (P), resin modified glass ionomer cement (K), calcium aluminate glass ionomer cement (C), glass ionomer cement (G)). One hundred and eighty dentin surfaces of the extracted molar teeth placed on acrylic cylinders were divided into 12 groups randomly (*n* = 15). Each cement group was further divided into control (CP, CC, CK, CG), 6.5% Pa-rich GSE (P6.5, C6.5, K6.5, G6.5) and 12.5% Pa-rich GSE (P12.5, C12.5, K12.5, G12.5) subgroups. In accordance with the manufacturer’s recommendations the cements were applied. After shear bond tests, surfaces were examined under a stereomicroscope. Median shear bond strength (in MPa) of CP, CK, CC, CG groups were 14.13, 7.05, 4.87, 3.86; for the P6.5, G6.5, C6.5, K6.5 groups they were 13.98, 13.42, 6.21, 3.27; and for the P12.5, C12.5, K12.5, G12.5 groups they were 15.08, 5.40, 3.10, 0.00, respectively. CK and K6.5 groups showed a significant difference from the K12.5 group (*p* < 0.05). Also, CG, G6.5 and G12.5 groups were found statistically different from each other (*p* < 0.05). Applied to the dentin surface, 6.5% Pa-rich GSE enhanced the bond strength of glass ionomer cements.

## 1. Introduction

Since the introduction of zinc phosphate cements in 1879, cements for prosthodontic restorations advanced and application techniques developed. Polycarboxylate cement can be seen as an alternative to phosphate cement because it presents strong interlocking to dentin and lower acidity at early stages. Glass ionomer cements are also among the alternatives due to the release of fluoride properties. Resin modified glass ionomer cements minimize complications such as cohesive failure due to the improved physical properties, in addition to the adhesion and fluoride release properties of conventional glass ionomer cements. In order to overcome these disadvantages such as solubility and lack of adhesion, resin composite cements were coupled with bonding agents [1].

The esthetic of restoration evolved with respect to the patient’s demands, and it is well-documented that cements may influence the outcome’s aesthetic success. It is of paramount importance to use a minimally invasive approach within dentistry. This approach created a new demand in the use of restorative properties at the micro- and macro-level, and this perspective also had an effect on bioactive cements. A bioactive material is defined as a substance that results in the formation of an apatite-like material as a surface layer in the presence of a simulated body fluid [2].

A glass ionomer-modified bioceramic cement that is a water-based hybrid composition including calcium aluminate and glass ionomer, was mixed with distilled water. It sets through an acid-base reaction, similar to traditional glass ionomer cement adhesive reaction. The material is slightly acidic (pH 4) immediately after setting, whilst it neutralizes 1 h after setting. Three or four h later, the material presents a basic pH of 8.5 which throughout its service [1].

To consider cement as a bioactive material, it has to form apatite on the surface while in contact with phosphate-containing solutions [3]. Providing the basic pH is crucial for the biocompatibility profile of the material. In addition, the material overproduces Ca^2+^ ions that contribute to bioactivity. The calcium aluminate improves the glass ionomer cement structure and prevents the ionomer from continuously leaking over time. This material has an initial fluoride release comparable to the glass ionomer, but the release decreases over time [1].

As a result of a study done by Engstrand et al., calcium aluminate-based dental cement has been shown to provide a good environment for the formation of hydroxyapatite (HA). Furthermore, it has been indicated that human saliva contains sufficient amounts of calcium and phosphate ions to trigger the development of HA on the cement surface [4].

In a study conducted by Zmener et al., a comparison was made with a resin modified glass ionomer cement (Rely X Luting Plus) and a glass ionomer cement (Ketac ™ Cem) to determine the sealing properties of the bioactive material. No statistically significant difference in bacterial leakage between bioactive material and Rely X Luting Plus was found [5].

Adhesive restorations are preferred for treatment in many cases such as decayed and fractured tooth tissue. Adhesive interlocking of the cements to the dentin surface is very important both for the protection of the dental tissue and for the longevity of the restoration.

Although adhesive systems have significantly improved, the bonded interface including the mixture of dentin organic matrix, residual hydroxyapatite crystals and adhesive agents, is still the weakest region of adhesive restorations [6].

In addition, components of endogenous proteolytic enzymes, oral fluid and bacterial products are also involved in the degradation of the bonded interface [7].

Type I collagen fibrils constitute 90% of the dentin organic matrix. The remaining 10% are non-collagenous proteins such as proteoglycans and phosphoproteins [8]. By stimulating exogenous collagen cross-links, it is predicted that mechanical stability will increase and the rate of biodegradation of collagen will be reduced [9].

Synthetic agents such as glutaraldehyde and natural occurrences such as genipin and proanthocyanidin have been reported to induce exogenous collagen cross-links [10].

A natural collagen cross-linker, proanthocyanidin, can easily precipitate proline-rich proteins such as collagen because of its hydrogen and covalent bonds [10].

Grape seed and cocoa are the richest sources of proanthocyanidin, as well as many flowers, fruits, nuts, seeds and vegetables. Proanthocyanidin-rich grape seed extract (Pa-rich GSE) has been shown to improve the mechanical properties of demineralized dentin [11].

This study aimed to evaluate the effect of a new extract, Pa-rich GSE, at different concentrations (6.5% and 12.5% m/wt) on the bond strength of different cements. The null hypothesis is that there will be no influence of Pa-rich GSE on the bond strength of different cements and there will be no difference between groups.

## 2. Material and Methods

### 2.1. Preparation of Pa-Rich GSEs

**Figure 1:**
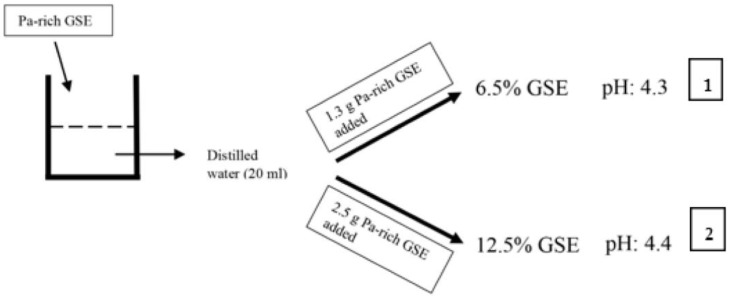
Experimental setup of two different concentrations of proanthocyanidin extract. Pa-rich GSE: proanthocyanidin-rich grape seed extract.

Two different concentrations of grape seed extract (GSE) were prepared for this study (Figure 1).

(1) 6.5% GSE: 6.5% GSE solution was prepared by adding 1.3 g Pa-rich GSE (>95%, Oligomeric Proanthocyanidin, Indena S.p.A., Milan, Italy) to distilled water (20 ml). The pH 4.3 for 6.5% GSE solution was recorded by pH meter (Seven Easy, Mettler Toledo, Switzerland).
(2) 12.5% GSE: 12.5% GSE solution was prepared by adding 2.5 g Pa-rich GSE (>95%, Oligomeric Proanthocyanidin, Indena S.p.A., Milan, Italy) to distilled water (20 ml). The pH of the 12.5% GSE solution was 4.4 (pH meter: Seven Easy, Mettler Toledo, Switzerland).

### 2.2. Preparation of Dentin Specimens

A total of 180 dentin specimens *(n =* 15 per group; 12 groups (four different cement materials, extracted proanthocyanidin at two different concentrations, one control)) were prepared from extracted human molar teeth (Near East University’s Ethics Review Board-Ethical Approval no: 632-2018). The molars were kept in distilled water after being cleaned. For the preparation of the samples, the teeth were placed centrally in cylinders (20 × 25 mm) and cold acrylic (Heracus Kulzer Ltd, Newbury, London) was poured. The occlusal surface was placed parallel to the base of the acrylic block. The occlusal surface of each sample was trimmed to the level below the occlusal pit and fissure and perpendicular to the long axis of the tooth with a diamond blade saw (Precision Sectioning Saw, Isomer 1000, Buehler, IL, USA). Then, the dentin surfaces were polished with 220-, 400-, and 600-grit SiC papers (ZiBo Sisho MT Coated Abrasive CO, Ltd, Shandong, China) to create a smooth surface under water-cooling [11].

### 2.3. Preparation of Shear Bond Strength Specimens

Fifteen teeth were selected randomly from the 180 teeth for four different cement groups (P, C, K, G). For the control groups (CP, CC, CK, CG) Pa-rich GSE was not applied onto the dentin. However, for 6.5% (P6.5, C6.5, K6.5, G6.5) and 12.5% (P12.5, C12.5, K12.5, G12.5) Pa-rich GSE groups, the 6.5% and 12.5% Pa-rich GSEs were applied onto the dentin for 10 minutes. A glass ionomer cement (GC Fuji 1 Capsule, GC Corporation, Tokyo, Japan (G)), a resin modified glass ionomer (Ketac Cem Plus, 3M, ESPE, St. Paul, MN, USA (K)), a resin cement (Panavia V5, Kuraray Noritake Dental, Kurashiki, Japan (P)), and a glass ionomer-modified bioceramic cement (Ceramir Crown & Bridge QuikCap, Doxa Dental AB, Uppsala, Sweden (C)) were applied to the dentin surfaces and cured according to the manufacturer’s recommendations (Table 1). The test set up used the Ultradent shear bond strength method by using molds with 2.8 mm. The resin, glass ionomer and resin modified glass ionomer groups were immersed in the humidified environment for 24 h at 37 °C, and the glass ionomer-modified bioceramic groups were stored in sterile phosphate buffer solution (Sigma-Dulbecco’s Phosphate Buffered Saline, Sigma-Aldrich, St. Louis, MO, USA) at 37 °C for 24 h.

### 2.4. Shear Bond Strength Testing and Failure Mode Determination

The shear bond strength (in MPa) was measured with a universal test machine with preload 5.00 N and crosshead-speed of 0.750 mm/min (EZ-test 500 N Shimadzu, Kyoto, Japan) in a software program (Nexygen Software, Lloyd Inc, Leicester, UK). Connection failure zones were visually examined under a stereomicroscope (Leica S8 APO; Leica Microsystems GmbH, Wetzlar, Germany) at 40× magnification to determine fracture modes. The fracture mode for each sample was observed and classified in one of the three categories given below:

- Adhesive
- Cohesive
- Adhesive and cohesive (mix)

### 2.5. Statistical Analysis

Analyses were performed by using a statistical software (SPSS 24 software, SPSS Inc., Chicago, IL, USA). In order to analyze possible differences in bond strength regarding the levels of each independent variable, the Kruskal-Wallis test was used. The Mann–Whitney U test was carried out to compare the groups bilaterally and Bonferroni correction was used. Friedman test procedures were used to observe the differences in bond strength score at different concentrations. A 4 × 3 factorial ANOVA procedure for non-normal distributions was used to test for possible interaction effects. In order to understand the strength of the relationship between variables and the magnitude of differences, η² (partial eta square) effect size measures were used (α = 0.05).

## 3. Results

The data were not normally distributed; therefore, we applied the Kruskal–Wallis analysis of variance test to compare between groups. Whenever the differences were statistically significant we compared groups bilaterally using the Mann–Whitney U test (Bonferroni correction was used too).

Median, minimum (min), maximum (max) values, and interquartile range (IQR) of different cements with different concentrations of Pa-rich GSE are summarized in Table 2.

As a result of statistical analysis between the control groups of different cements, CP group is significantly different from CK (*p* = 0.025), CC (*p* = 0.000), and CG (*p* = 0.000) groups (Table 2). On the other hand, median bond strength score of the CK group is greater than the median score of CG group (*p* = 0.014) (Table 2). There was no evidence of a difference between the other pairs (*p* > 0.05).

According to the statistical results of different cements with 6.5% Pa-rich GSE, the median bond strength score for the P6.5 group is greater than the median bond strength scores of the K6.5 (*p* = 0.000) and C6.5 (*p* = 0.010) groups (Table 2). There is no significant median bond strength score between P6.5 and G6.5 groups (*p* > 0.05). Median bond strength score of G6.5 group is significantly greater than K6.5 (*p* = 0.000) and C6.5 (*p* = 0.029) groups. There was no evidence of a difference between the other pairs (*p* > 0.05).

As a result of statistical analysis between the 12.5% Pa-rich GSE and different cement groups, the median bond strength score for the P12.5 group is greater than median bond strength scores of the K12.5 *(p* = 0.001), C12.5 (*p* = 0.001) and G12.5 (*p* = 0.000) groups (Table 2). There was no evidence of a difference between the other pairs (*p* > 0.05).

The data are adjusted by subtracting the marginal mean from each relevant observation to analyze interaction effects. In line with this a 4 × 3 factorial ANOVA on the adjusted ranked bond strength scores was conducted. A significant interaction (*p* = 0.000) means that the bonding strength differs significantly regarding different cements when using different concentrations (Table 3).

**Figure 2:**
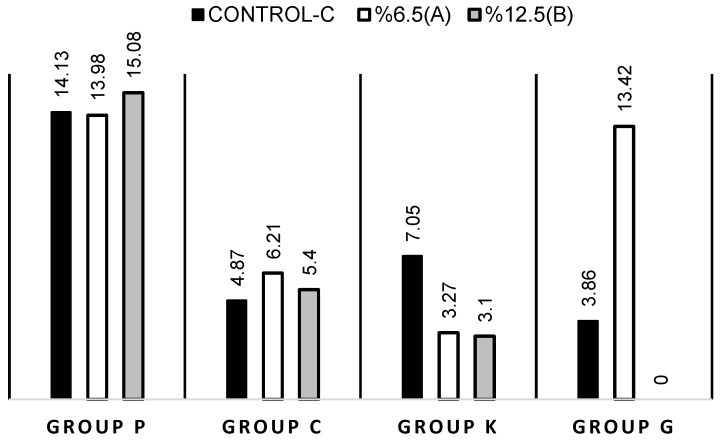
Median scores for different cements at different concentrations.

In accordance with Friedman test results, it was revealed that there are no significant differences among the mean ranks of P for different concentrations, χ^2^ (2, *n* = 15) = 1.2, *p =* 0.549 (Figure 2). Similarly, it was revealed that there are no significant differences among the mean ranks of C for different concentrations, χ^2^ (2, *n* = 15) = 2.133, *p =* 0.344 (Figure 2) and there are significant differences among the mean ranks of K for different concentrations, χ^2^ (2, *n* = 15) = 15.6, *p =* 0.000 favoring the control group, *p* ≤ 0.019 (Figure 2). Friedman test results revealed that there are significant differences among the mean ranks of G for different concentrations, χ^2^ (2, *n* = 15) = 14.93, *p =* 0.001. Median bond strength score of G6.5 group is greater than the median bond strength score of G12.5 group (*p* = 0.001) and with CG, *p* = 0.010 (Figure 2). There is no significant difference of bond strength medians of CG and G12.5 group (*p* > 0.05).

**Figure 3:**
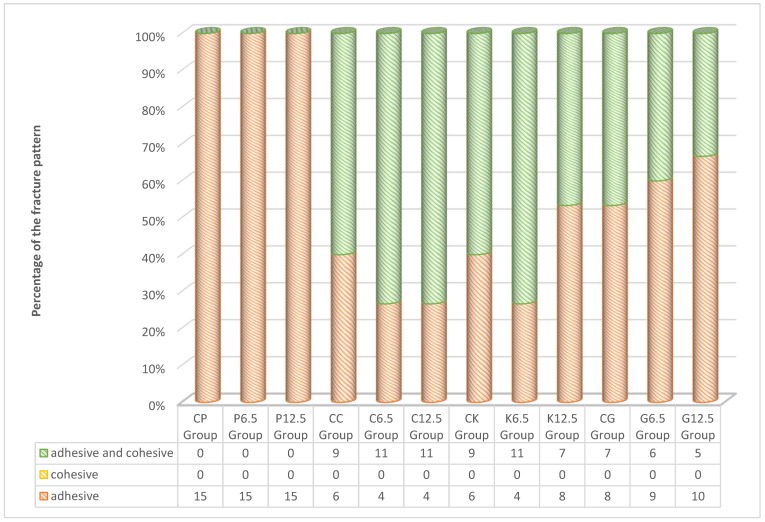
Failure modes of groups after the shear bond strength test.

Failure modes of specimens (in percentage) after the shear bond strength test (Figure 3). Adhesive-type and mixed-type fractures are the most common types, as seen in the present study. In Ceramir groups, different from its control group (C6.5 and C12.5), an increasing mix failure rate was observed. The same trend was observed for only K6.5 group of Ketac. For G groups (G6.5 and G 12.5) a decreasing mix failure rate was observed.

## 4. Discussion

As the bond strength of G group enhanced after the application of 6.5% Pa-rich GSE, the null hypothesis was rejected. The null hypothesis was rejected because CP group showed higher dentin bond strength than the other control groups (CK, CC, CG); P6.5 group showed higher bond strength than K6.5 and C6.5, and G6.5 group also showed greater values then K6.5 and C6.5 groups. In addition to these results, P12.5 group showed higher dentin bond strength then K12.5, C12.5 and G12.5 groups.

In general, according to bond strength results, cements are ascendingly ranked as zinc phosphate cement, glass ionomer cement, resin modified glass ionomer cement and resin luting cement. This tendency may be related to the intrinsic strength of the cement. As the resin content increases, strength increases [12,13]. Various mechanical tests have been conducted in order to find the bonding performance of luting materials [14]. To find out the bond strength of cements, shear and tensile tests which need less equipment and sample preparation, are widely used because of their advantages [15]. According to many researches, shear testing has been generally used to figure out the bonding strength of cements to tooth dentin [16,17]. Interfacial stress, which occurs during the specimen preparation, causes failure before the shear bond tests, especially for susceptible materials [18]. Glass ionomer cement shows low bonding performance, so the shear bond testing is less complicated to apply onto the dentin surface [19]. Therefore, this study was conducted in vitro to evaluate the shear bond strength of various cements combined with different concentrations of Pa-rich GSE to dentin.

Proanthocyanidin is a naturally occurring compound which interacts with proteins to induce cross-linking through four different mechanisms. These mechanisms include covalent interaction, ionic interaction, hydrogen bonding and hydrophobic interaction [20,21,22]. In agreement with Macedo et al.’s study, the effect of proanthocyanidin on dentin surfaces could be described by attribution to the specificity of proanthocyanidin to induce the enzyme proline hydroxylase which catalyzes the hydroxylation of proline as an essential step in collagen biosynthesis [23,24,25]. Therefore, proanthocyanidin is more capable of interacting with collagen than other cross-linking agents [9].

According to our results Panavia with 6.5% Pa-rich GSE group showed significantly higher bond strength to dentin compared to Ketac with 6.5% Pa-rich GSE and Ceramir with 6.5% Pa-rich GSE group. This is in accordance with the findings of Srinivasulu et al.’s study which showed that the application of 6.5% Pa-rich GSE to deep dentin significantly improved the shear bond strength values of composite to dentin compared with the use of 10% sodium ascorbate. The highest bond strength of P group may be explained with the greater number of collagen cross-links which improved collagen stability. Also, among the experimental groups, the highest adhesion was observed in the P group. This high rate of adhesion was based on the amine-free redox initiator mechanism of P [26]. For this study the main reason for P preference was due to simplified steps of manipulation. Also, using “touch and cure” systems in P is important to enhance dentin bonding performance. The polymerization reaction starts by mixing base and catalyst paste, thus it chemically activates the initiator. Photo initiation allows the advancement of the polymerization reaction until a restoration is correctly placed. Then, excess cement is removed [27]. Furthermore, higher adhesion of dentin tissue to P unlike other materials may be related with the presence of methacryloyloxdecyl dihydrogen phosphate in the primary content of the material [28]. With another mechanism, this high adhesive bonding of P group may correlate with its low water sorption [29], fluoride realizing property and durability [30].

The clinical bioactivity of Ceramir has been reported by Jefferies et al. [31,32]. In addition, allowing hydroxyapatite formation and a long-term sealing between the tooth and the cement interface can be attributed to the similar values obtained at both Pa-rich GSE concentrations of Ceramir (6.5%, 12.5%) compared to the control group in the present study.

Dental cements present a link between the intaglio surface of the restoration and prepared tooth surface, bonding them together through a number of bonding mechanisms, which can be micro-mechanical, chemical or a combination. Glass ionomer cement adheres to enamel and dentin tissues due to the presence of polyacrylic acid in the liquid and therefore, provides a chemical link between restoration and the prepared tooth. After 24 h it exhibits compressive and tensile bond strength values comparable with zinc phosphate cement [33]. In the present study, a bond strength of 3.86 MPa for glass ionomer cement was detected. This is in accordance with a study by Michelini et al. [34]. The shear bond strength of glass ionomer cement is low because of its low flexural strength and compressive strength. Resin modified glass ionomer cement was employed to improve this characteristic of cements. This cement presents a higher bond strength of 12.25 MPa to dentin, compared to glass ionomer and zinc phosphate cement. With the development of new molecules, an unsaturated hydrophilic polymer, the strength was improved [33]. The study by Almuammar et al. [35] has shown that the bonding resistances of the resin modified glass ionomer cements are higher than conventional glass ionomer cements. This study also confirms the same results.

In the present study, CP group showed significantly higher bond strength values to dentin compared with CK, CC and CG groups. This is accordance with the findings of a study by Mitchell et al., since the bond strength of glass ionomer cement is much lower than those of resin modified glass ionomer cement, which has a lower strength than resin composite cement [36]. This fact is reflected in the shear bond strength value of the different bonding cements tested in this study.

In our study, application of Pa-rich GSE at different concentrations (6.5%, 12.5%) to Panavia cement did not affect the resin-dentin bond strength. However, opposite results were also reported in a study that showed the use of 6.5% proanthocyanidin as a collagen cross-linker on the deep dentin, significantly improving the shear bond strength values [37].

As a description of the limitations of the study’s in-vitro conditions does not simulate oral cavity, only four cements were used in this study. Different cements may provide different results. Only the influence of grape seed extract in different concentrations was investigated. Different cross-linkers (sodium ascorbate, riboflavin/chitosan modification) may alter the outcome.

In the light of this study, limited information regarding resin modified glass ionomer, glass ionomer and calcium aluminate glass ionomer cement is available in the literature, and further studies are needed.

## 5. Conclusions

The following conclusions can be drawn within the limitations of this study:

1. Resin cement showed higher bond strength to dentin than other cements.

2. The application of the extract containing 6.5% proanthocyanidin to dentin surfaces increased the bonding of conventional glass ionomer cement to dentin.

3. While there was no difference in dentin–cement bonding between resin and calcium aluminate glass ionomer cement by applying 12.5% Pa-rich GSE to dentin surfaces, resin modified, and conventional glass ionomer cement weakened dentin bonding.

4. Calcium aluminate glass ionomer cement showed similar shear bond strength values with other cements except resin cement.

According to these results, as an alternative to resin modified glass ionomer cement and glass ionomer cement, calcium aluminate glass ionomer cement can be tried in clinical studies, and more studies can be done with glass ionomer cements and 6.5% Pa-rich GSE.

As a result, we may affirm that the first variable for changes in shear bond strengths is type of material. However, surface preparation with 6.5% Pa-rich GSE could increase the shear bond strength of less resistant material like conventional glass ionomer cement.

## Figures and Tables

**Table 1:** Types and compositions of the cements used in the study.

Materials	Types	Composition
GC Fuji 1 Capsule	Glass ionomer cement	Powder: fluoroaluminosilicate glass Liquid: polyacrylic acid
Ketac Cem Plus	Resin-modified glass ionomer cement	Powder: fluoroaluminosilicate glass Liquid: methacrylated polyacrylic acid
Ceramir C&B QuickCap	Hybrid Calcium Aluminate glass ionomer cement	Tartaric acid Strontium fluoride Poly(acrylic acid) Acid soluble glass (SiO_2_-Al_2_O_3_-SrO-P_2_O_5_-NaO_2_-F^−^) Ground calcium aluminate
Panavia V5	Dual-cure resin cement	Tooth primer: 10-MDP, HEMA, Hydrophilic aliphatic dimethacrylate, Accelerators, water Cement: Bis-GMA, TEGDMA, Hydrophobic aromatic dimethacrylate, hydrophilic aliphatic dimethacrylate, Initiators, Accelerators, Silanated barium glass filler, Silanated fluoroaluminosilicate glass filler, Colloidal silica, Silanated aluminium oxide filler, dl Camphorquinone, Pigments

10-MDP: 10-methacryloyloxydecyl dihydrogen phosphate; HEMA: hydroxyethyl methacrylate; Bis-GMA: bisphenol-A-diglycidylmethacrylate; TEGDMA: triethylene glycol dimethacrylate.

**Table 2:** Median, minimum (min), maximum (max) values, and interquartile ranges (IQR) of different cements with different concentrations of Pa-rich GSE.

		P	C	K	G
**Control** **(C)**	Median (min–max)	14.13 ^b,c,d^ (6.32–22.37)	4.87 ^a^ (2.83–8.50)	7.05 ^a,d,B,C^ (4.49–8.06)	3.86 ^a,B^ (1.34–6.96)
	IQR	5.42	2.70	1.00	2.81
**6.5% Pa-rich GSE (6.5)**	Median (min–max)	13.98 ^b,c^ (4.65–16.81)	6.21 ^a,d^ (3.29–9.60)	3.27 ^a,d,A,C^ (1.64–6.07)	13.42 ^b,c,A,C^ (5.29–25.33)
	IQR	5.62	3.23	3.03	12.45
**12.5% Pa-rich GSE (12.5)**	Median (min–max)	15.08 ^b,c^ (6.98–24.74)	5.40 ^a,d^ (1.18–7.89)	3.10 ^a,d,A,B^ (1.36–6.99)	0.00 ^b,c,B^ (0.00–32.11)
	IQR	11.10	3.69	4.92	7.21

Superscript letters in the same row: a—significant difference from cement P. b—significant difference from cement C. c—significant difference from cement K. d—significant difference from cement G. (*p* < 0.05). Superscript letters in the same column: A—significant difference from group control. B—significant difference from group 6.5% Pa-rich GSE. C—significant difference from group 12.5% Pa-rich GSE.

**Table 3:** Factorial ANOVA results.

Source	*SS*	*df*	*F*	*η* ^2^	*p*
Interaction 6.5 × 12.5	85,878.37	6	12.424	0.307	0.000
Error	193,545.6	168			
Total	1,960,230.0	180			

## References

[1] Pameijer C.H. (2012). A review of luting agents. Int. J. Dent..

[2] Jefferies S.R., Fuller A.E., Boston D.W. (2015). Preliminary evidence that bioactive cements occlude artificial marginal gaps. J. Esthet. Restor. Dent..

[3] Lööf J., Svahn F., Jarmar T., Engqvist H., Pameijer C.H. (2008). A comparative study of the bioactivity of three materials for dental applications. Dent. Mater..

[4] Engstrand J., Unosson E., Engqvist H. (2012). Hydroxyapatite formation on a novel dental cement in human saliva. ISRN Dent..

[5] Zmener O., Pameijer C.H., Rincon S.M.H., Serrano S.A., Chaves C. (2013). Sealing properties of three luting agents used for complete cast crowns: A bacterial leakage study. Oper. Dent..

[6] Castellan C.S., Pereira P.N., Grande R.H.M., Bedran-Russo A.K. (2010). Mechanical characterization of proanthocyanidin–dentin matrix interaction. Dent. Mater..

[7] Hashimoto M., Ohno H., Sano H., Kaga M., Oguchi H. (2003). In vitro degradation of resin–dentin bonds analyzed by microtensile bond test, scanning and transmission electron microscopy. Biomaterials.

[8] Goldberg M., Kulkarni A.B., Young M., Boskey A. (2011). Dentin: Structure, Composition and Mineralization: The role of dentin ECM in dentin formation and mineralization. Front. Biosci. (Elite Ed.).

[9] Al-Ammar A., Drummond J.L., Bedran-Russo A.K. (2009). The use of collagen cross-linking agents to enhance dentin bond strength. J. Biomed. Mater. Res. B.

[10] Tang C.F., Fang M., Liu R.R., Dou Q., Chai Z.G., Xiao Y.H., Chen J.H. (2013). The role of grape seed extract in the remineralization of demineralized dentine: Micromorphological and physical analyses. Arch. Oral Biol..

[11] Odthon P., Khongkhunthian P., Sirikulrat K., Boonruanga C., Sirikulrat N. (2015). In vitro shear bond strength test and failure mechanism of zinc phosphate dental cement. Int. J. Adhes. Adhes..

[12] Al–Makramani B.M., Razak A.A., Abu–Hassan M.I., Al–Sanabani F.A., Albakri F.M. (2018). Effect of Luting Cements on the Bond Strength to Turkom-Cera All-Ceramic Material. Open Access Maced. J. Med. Sci..

[13] Özcan M., Alkumru H.N., Gemalmaz D. (2001). The effect of surface treatment on the shear bond strength of luting cement to a glass-infiltrated alumina ceramic. Int. J. Prosthodont..

[14] Poggio C., Beltrami R., Scribante A., Colombo M., Lombardini M. (2014). Effects of dentin surface treatments on shear bond strength of glass-ionomer cements. Ann. Stomatol..

[15] Öztürk E., Bolay Ş., Hickel R., Ilie N. (2013). Shear bond strength of porcelain laminate veneers to enamel, dentine and enamel–dentine complex bonded with different adhesive luting systems. J. Dent..

[16] Shehata W.K., Aziz A.E., A El-Naggar G., Abdel Ghany O.S. (2018). Effect of Sandblasting and Zirconia Primer Application on The Zirconia-Cement Shear Bond Strength (An in-vitro Study). Al-Azhar Dent. J. Girls.

[17] Schmidt A., Schäfer E., Dammaschke T. (2017). Shear bond strength of lining materials to calcium silicate cements at different time intervals. J. Adhes. Dent..

[18] Salz U., Bock T. (2010). Testing adhesion of direct restoratives to dental hard tissue—A review. J. Adhes. Dent..

[19] Wang L., Sakai V.T., Kawai E.S., Buzalaf M.A.R., Atta M.T. (2006). Effect of adhesive systems associated with resin-modified glassionomer cements. J. Oral Rehabil..

[20] Nagpal R., Singh P., Singh S., Tyagi S.P. (2016). Proanthocyanidin: A natural dentin biomodifier in adhesive dentistry. J. Restor. Dent..

[21] Xu L.Q., Neoh K.G., Kang E.T. (2018). Natural polyphenols as versatile platforms for material engineering and surface functionalization. Prog. Polym. Sci..

[22] Feiz A., Badrian H., Goroohi H., Mojtahedi N. (2017). The Effect of Synthetic Grape Seed Extract (GSE) on the Shear Bond Strength of composite resin to Dentin. J. Res. Med. Dent. Sci..

[23] Macedo G., Yamauchi M., Bedran-Russo A. (2009). Effects of chemical cross-linkers on cariesaffected dentin bonding. J. Dent. Res..

[24] Ku C.S., Sathishkumar M., Mun S.P. (2007). Binding affinity of proanthocyanidin from waste Pinus radiata bark onto proline-rich bovine achilles tendon collagen type I. Chemosphere.

[25] Gajjela R.S., Satish R.K., Sajjan G.S., Varma K.M., Rambabu T., Lakshmi B.V. (2017). Comparative evaluation of chlorhexidine, grape seed extract, riboflavin/chitosan modification on microtensile bond strength of composite resin to dentin after polymerase chain reaction thermocycling: An in vitro study. J. Conserv. Dent. JCD.

[26] Tagami A., Takahashi R., Nikaido T., Tagami J. (2017). The effect of curing conditions on the dentin bond strength of two dual-cure resin cements. J. Prosthodont. Res..

[27] Halabi S., Sato T., Ikeda M., Nikaido T., Burrow M.F., Tagami J. (2019). Adhesion durability of dual-cure resin cements and acid–base resistant zone formation on human dentin. Dent. Mater..

[28] Rohr N., Fischer J. (2017). Tooth surface treatment strategies for adhesive cementation. J. Adv. Prosthodont..

[29] Müller J.A., Rohr N., Fischer J. (2017). Evaluation of ISO 4049: Watersorption and water solubility of resin cements. Eur. J. Oral Sci..

[30] Yoshida Y., Nagakane K., Fukuda R., Nakayama Y., Okazaki M., Shintani H. (2004). Comparative study on adhesiveperformance of functional monomers. J. Dent. Res..

[31] Jefferies S.R., Pameijer C.H., Appleby D., Boston D., Lööf J., Glantz P.O. (2009). One year clinical performance and post-operative sensitivity of a bioactive dental luting cement. Swed. Dent. J..

[32] Jefferies S.R., Pameijer C.H., Appleby D.C., Boston D., Galbraith C., Lööf J., Glantz P.O. (2012). Prospective Observation of a New Bioactive Luting Cement: 2-Year Follow-Up. J. Prosthodont..

[33] Kulkarni G. (2016). Comparative Analysis of Tensile Bond Strength of the Adhesive Luting Agents for a Nonpercious Alloy (Ceramo-Metal) to Dentin: An In Vitro Study. J. Int. Oral Health.

[34] Michelini F.S., Scherrer S.S., De Rijk W.G. (1995). Tensile bond strength of gold and porcelain inlays to extracted teeth using three cements. Int. J. Prosthodont..

[35] Almuammar M.F., Schulman A., Salama F. (2001). Shear bond strength of six restorative materials. J. Clin. Pediatr. Dent..

[36] Mitchell C.A., Abbariki M., Orr J.F. (2000). The influence of luting on the probabilities of survival and modes of failure of cast fullcoverage crowns. Dent. Mater..

[37] Srinivasulu S., Vidhya S., Sujatha M., Mahalaxmi S. (2013). Effect of collagen cross-linkers on the shear bond strength of a self-etch adhesive system to deep dentin. J. Conserv. Dent..

